# Natural Products from Mangrove Actinomycetes

**DOI:** 10.3390/md12052590

**Published:** 2014-05-02

**Authors:** Dong-Bo Xu, Wan-Wan Ye, Ying Han, Zi-Xin Deng, Kui Hong

**Affiliations:** Key Laboratory of Combinatorial Biosynthesis and Drug Discovery, Ministry of Education, School of Pharmaceutical Sciences, Wuhan University, Wuhan 430071, China; E-Mails: xudongbo@whu.edu.cn (D.-B.X.); yy_ww1001@163.com (W.-W.Y.); hanying0928@whu.edu.cn (Y.H.); zxdeng@whu.edu.cn (Z.-X.D.)

**Keywords:** mangrove actinomycetes, natural products, bioactivity, drugs discovery

## Abstract

Mangroves are woody plants located in tropical and subtropical intertidal coastal regions. The mangrove ecosystem is becoming a hot spot for natural product discovery and bioactivity survey. Diverse mangrove actinomycetes as promising and productive sources are worth being explored and uncovered. At the time of writing, we report 73 novel compounds and 49 known compounds isolated from mangrove actinomycetes including alkaloids, benzene derivatives, cyclopentenone derivatives, dilactones, macrolides, 2-pyranones and sesquiterpenes. Attractive structures such as salinosporamides, xiamycins and novel indolocarbazoles are highlighted. Many exciting compounds have been proven as potential new antibiotics, antitumor and antiviral agents, anti-fibrotic agents and antioxidants. Furthermore, some of their biosynthetic pathways have also been revealed. This review is an attempt to consolidate and summarize the past and the latest studies on mangrove actinomycetes natural product discovery and to draw attention to their immense potential as novel and bioactive compounds for marine drugs discovery.

## 1. Introduction

In the past few decades, repeated isolation of known natural products and a reduced hit-rate of novel compounds from heavily investigated terrestrial microorganisms have limited the development of new and effective drugs for treating ever increasing human diseases [1]. On the other hand, the arising antibiotic-resistant pathogens present an urgent requirement for new bioactive compounds discovery. Hence, more and more researchers have switched over to new or extreme environments for novel pharmaceutical compounds such as deep oceans [2,3], deserts [4], polar areas [5] and mangroves [6]. The mangrove ecosystem has made its way into the researchers’ view because of its special ecosystem between sea and land, the superiority of easy sampling and the rich biological diversity.

Global mangroves are mainly distributed in Asia (42%), Africa (20%), North and Central America (15%), Oceania (12%) and South America (11%) [7], and cover about 60%–75% of the global tropical and subtropical coastlines [8]. Mangrove is a high moisture, high salinity, and hypoxia tolerant ecosystem [9] which breeds many kinds of novel microorganisms and plants that have been a rich source of bioactive natural products [10,11,12,13]. Mangrove trees have been acknowledged as medicinal plants by the local people. About 70 mangrove plant species of 27 genera have been reported and extracts from these plants showed antimicrobial activities against human, animal and plant pathogens as well as antinociceptive, anti-inflammatory, and extensive antipyretic activity [14,15]. In addition, Wu *et al.* [10,16] summarized the source, chemistry and bioactivity of natural products from semi-mangrove and true-mangrove and revealed the great research value of mangrove plants. Calcul *et al.* [12] screened 5486 mangrove endophytic fungi for antimalarial natural products and four new compounds with high bioactivity were found. More than 200 species of mangrove endophytic fungi were isolated and natural products from them showed potential anti-microbial and anti-tumor activity [13]. Meanwhile, over 2000 actinomycetes were isolated from mangroves and their secondary metabolites showed anti-infection, anti-tumor and protein tyrosine phosphatase 1B (PTP1B) inhibitory activity [11].

Actinomycetes are known to be active and numerous components of marine microbial communities as well as diverse in various marine ecosystems including mangroves. This valuable microbial community produces different types of fascinating and structurally complex natural products with biological activity. Numerous researches have indicated that actinomycetes secondary metabolites have potential as new antibiotics, antitumor agents, immunosuppressive agents and enzyme inhibitors [17]. Up till now, about 23,000 bioactive compounds produced by microorganisms have been reported and more than 10,000 among these compounds were isolated from actinomycetes [18]. It is worth mentioning that of 10,000 compounds, about 80% have been obtained from *Streptomyces* which is the most productive genus in the microbial world [19].

Recently, bioactivity studies of secondary metabolites from mangrove actinomycetes have become a hot spot. The reported screening tests are not only on antimicrobial, antineoplastic and PTP1B inhibitory activity [11] but also on protease, cellulase, amylase, esterase, l-asparaginase production [20,21]. Compounds with unique structure and potential medicinal use have been obtained from mangrove actinomycetes in recent years. The well-known compound salinosporamide A, is the first and most advanced marine actinomycete secondary metabolite to be processed for clinical trials for cancer treatment, and was actually produced by the *Salinispora* strain isolated from a mangrove sample [22]. Other examples are the xiamycins, which are indolosesquiterpenes compounds, isolated from prokaryotes for the first time [23]. Novel indolocarbazoles identified from a mangrove streptomycete present unprecedented cyclic *N*-glycosidic linkages between 1,3-carbon atoms of the glycosyl moiety and two indole nitrogen atoms of the indolocarbazole core with anticancer activity [24]. The mangrove environment is a virtually untapped source of novel and diverse natural products and needs much more attention.

The mangrove environment such as geographical location, pH, temperature, salinity, moisture and nutrient differs greatly in different regions so that mangrove actinomycetes are diverse and unique [6]. So far, 24 genera of 11 families and eight suborders under the actinomycetale containing three new genera have been isolated and identified from mangrove. *Streptomyces* has the biggest advantage in the ability to produce bioactive compounds as well as isolation quantity [25]. However, it is difficult to define an individual species or genus as unique mangrove actinomycetes based on the evidence provided by detailed phylogenetic characterization of the actinomycete genus *Salinispora* that an individual species can be cosmopolitan in distribution when its growth requirements are met [26]. The limitation of studied bacterial populations and resolution of analytical techniques make it uncertain of detecting bacterial endemism. Even so, new species and genus in mangrove represent to a certain degree the uniqueness of mangrove actinomycetes and indeed produce novel bioactive compounds or chemical scaffolds.

In this review, we summarize the vast majority of studies about mangrove actinomycetes natural products and pharmacological activity and attempt to uncover the value of mangrove actinomycetes as an important source of novel and bioactive compounds which could become new and effective marine drugs.

## 2. Natural Products Produced by Mangrove Actinomycetes

### 2.1. Alkaloids

#### 2.1.1. Cyclic Dipeptides

**Figure 1:**
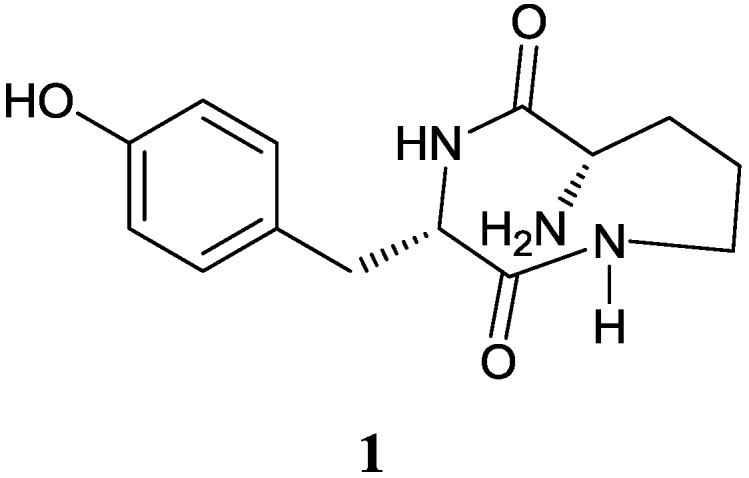
The structure of cyclic dipeptide (**1**).

A novel nitrogenous cyclic dipeptide (**1**) was isolated from the broth of *Streptomyces* sp. 124092 collected from rhizosphere soil of the mangrove plant *Heritiera littoralis* in Wenchang, Hainan, China and showed moderate cytotoxicity against SMMC-7721 with the IC_50_ value of 60 μg/mL [27]. The corresponding chemical structure is shown in Figure 1.

#### 2.1.2. Indole Alkaloids

Xiamycin (**2**) and its methyl ester (**4**) were discovered by Ding *et al.* [23] and obtained from *Streptomyces* sp. GT2002/1503, an endophyte from the mangrove plant *Bruguiera gymnorrhiza* in Xiamen, China. These indolosesquiterpene compounds were isolated from prokaryotes for the first time. Xiamycin showed selective anti-HIV activity which specifically blocked R5 tropic HIV-1 infection and its methyl ester was more active in antimicrobial, cytotoxic and antiviral biological assays. This research group then isolated another three novel indolosesquiterpenes, xiamycin B (**3**), indosespene (**5**), sespenine (**6**) and the known xiamycin A (**2**) from the culture broth of *Streptomyces* sp. HKI0595, an endophyte of the widespread mangrove tree *Kandelia candel* in Xiamen, Fujian Province, China [28]. These three new compounds have been proved to have strong antimicrobial activities against several bacteria, including methicillin-resistant *Staphylococcus aureus* and vancomycin-resistant *Enterococcus faecalis*. The same research group continued to unveil the molecular basis for the first indole terpenoid pathway in a bacterium and found two indispensable cyclases including XiaE and XiaF. The *xia* biosynthesis enzymes were believed to be a novel addition to the family of terpene synthases and modifying enzymes in bacteria [29]. In addition, Li *et al.* [30] isolated xiamycin A from a marine-derived *Streptomyces* sp. SCSIO 02999, and identified and characterized its gene cluster which revealed an oxidative cyclization strategy tailoring indolosesquiterpene biosynthesis. What is more, XiaM was identified as an enzyme capable of converting a methyl group to a carboxyl group via triple hydroxylations in xiamycin A biosynthesis [31].

**Figure 2:**
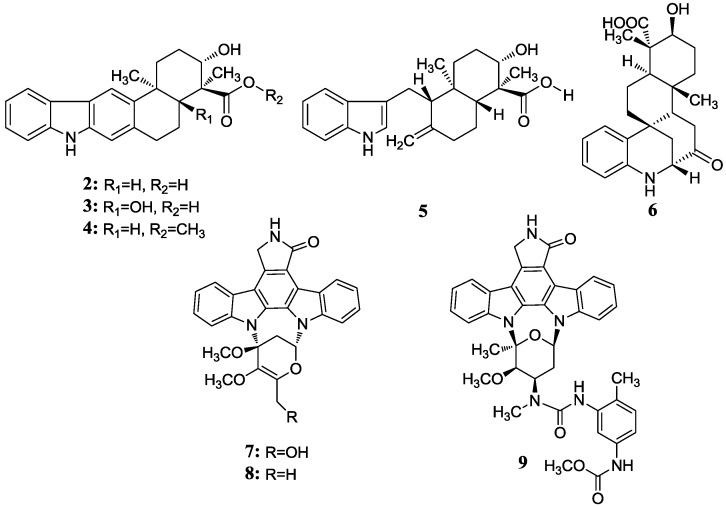
The structures of indole alkaloids (**2**–**9**).

Streptocarbazoles, novel staurosporine analogs with unprecedented cyclic *N*-glycosidic linkages between 1,3-carbon atoms of the glycosyl moiety and two indole nitrogen atoms of the indolocarbazole core were other promising compounds isolated from mangrove actinomycetes. Streptocarbazoles A (**7**) and B (**8**) were obtained from *Streptomyces* sp. FMA isolated from mangrove soil collected in Sanya, Hainan Province of China [24]. Compound **7** was active against HL60, A549, P338 and HeLa cells with IC_50_ values of 1.4, 5.0, 18.9 and 34.5 µM, respectively, while compound **8** was cytotoxic against P388 and HeLa cells with IC_50_ values of 12.8 and 22.5 μM, respectively. Streptocarbazoles A was proved to arrest the cell cycle of HeLa cells in the G_2_/M phase at a concentration of 10 μM. Recently, the first cloning and characterization of an indolocarbazole gene cluster isolated from *Streptomyces sanyensis* FMA was reported and indolocarbazole biosynthesis was confirmed by gene inactivation and heterologous expression in *Streptomyces coelicolor* M1152 [32]. In addition, Li *et al.* [33] also found a new staurosporine analog (**9**) which showed significant cytotoxicity against human colon tumor cell HCT-116 (IC_50_ = 0.37 µM) from the culture broth of *Streptomyces* sp. 172614 collected from mangrove soil in Jiulongjiangkou, Fujian, China. The corresponding chemical structures are shown in Figure 2.

#### 2.1.3. Naphthyridines

**Figure 3:**
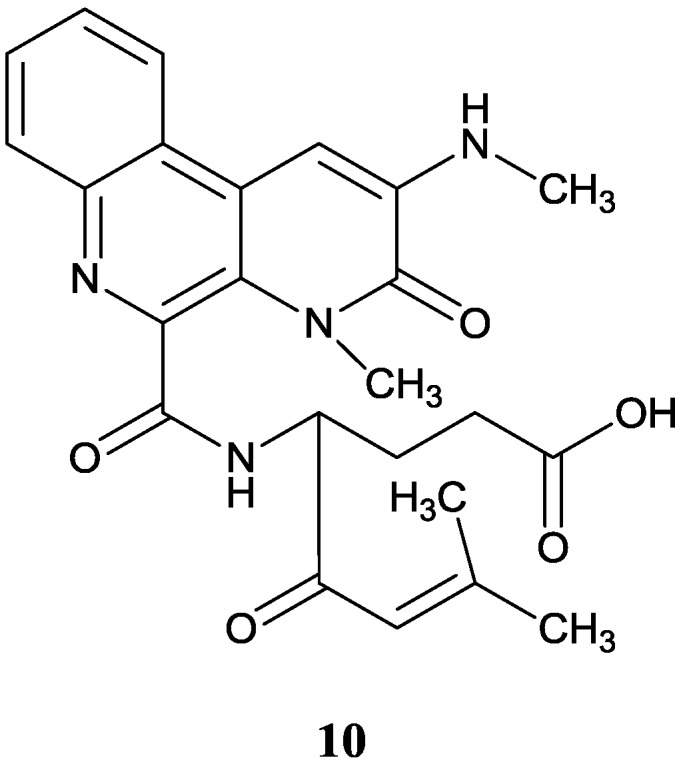
The structure of novel benzonaphthyridine alkaloid (**10**).

A novel alkaloid benzo[f][1,7] naphthyridine (**10**) was isolated from *Streptomyces albogriseolus* MGR072 originating from mangrove sediments collected in the national mangrove reserve in Fujian Province of China [34]. Further study was done by Tian *et al.* [35] who accomplished its first total synthesis and determined the absolute configuration and also proved it could inhibit HGC-27 (human stomach carcinoma cell line) moderately at a concentration of 45 μg/mL. The corresponding chemical structure is showed in Figure 3.

#### 2.1.4. Pyrazine Derivatives

**Figure 4:**
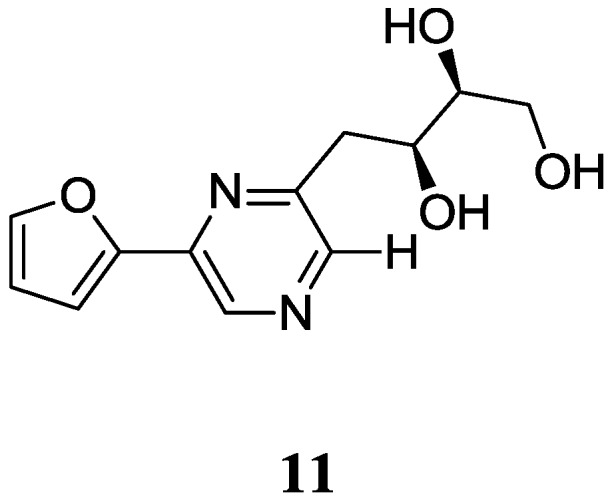
The structure of pyrazine derivative (**11**).

A new pyrazine derivative compound **11** was isolated from *Jishengella endophytica* 161111 [36], an endophytic actinomycetes which is a new genus of the Micromonosporaceae family [37]. This producer strain was isolated from a mangrove plant *Acanthus illicifolius* root in Hainan, China and compound **11** was proved to have no antivirus effects on H1N1. The corresponding chemical structure is shown in Figure 4.

#### 2.1.5. Salinosporamides

**Figure 5:**
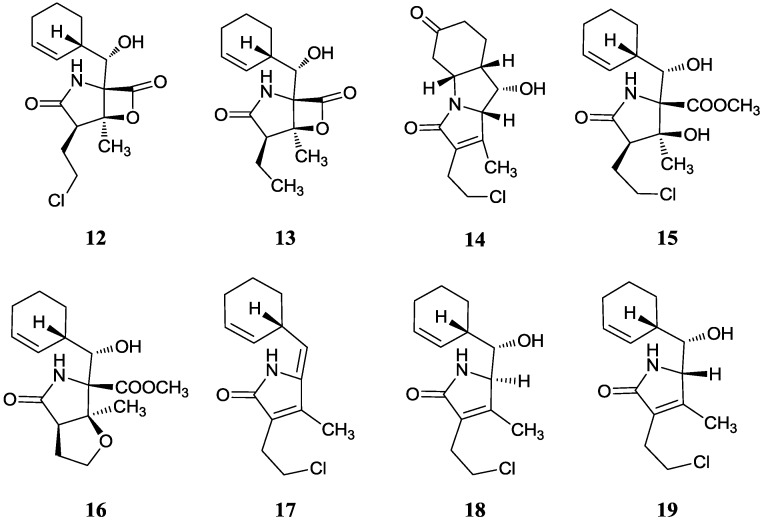
The structures of salinosporamides (**12**–**19**).

The best-studied secondary metabolite from mangrove actinomycetes is salinosporamide A (**12**) with γ-lactam-β-lactone bicyclic core. Feling *et al.* [22] reported it first in 2003 as a potent inhibitor of the 20S proteasome with an IC_50_ value of 1.3 nM from *Salinispora tropica* CNB-392 collected from a mangrove environment in Chub Cay, Bahamas, they then continued an extensive study of isolating seven other related γ-lactams (**13**–**19**) [38]. These compounds showed varying degrees of anticancer activities but no activity against *Staphylococcus aureus* (methicillin-resistant), *Enterococcus faecium* (vancomycin-resistant), and *Candida albicans* (wild-type and amphotericin-resistant) and *Herpes simplex* virus. In addition, they indicated that the chloroethyl moiety played a major role in the enhanced activity of salinosporamide A with a mean GI_50_ value of less than 10 nM in the NCI’s 60-cell-line panel. Then Liu *et al.* [39] reported the biosynthesis of salinosporamides from α,β-unsaturated fatty acids and discovered a new PKS extender unit propylmalonyl-CoA in the salinosporamide E biosynthesis. In addition total synthesis of salinosporamide A was reported by Endo and Danishefsky [40]. In 2010 Potts and Lam [41] carried out a review about the structures and activities of this class of compounds from fermentation and natural product chemistry, precursor-directed biosynthesis, mutasynthesis, semi-synthesis, and total synthesis. Then Potts *et al.* [42] gave a preclinical profile and a framework for clinical trials for Marizomib (salinosporamide A). The clinical trial of Marizomib in melanoma, pancreatic and lung cancer combined with the HDI (histone deacetylase inhibitor Vorinostat) showed highly synergistic antitumor activities [43]. Phase I trials in patients with advanced hematologic and solid malignancies also showed rapid, broad and effective proteasome inhibition with no significant PN, neutropenia or thrombocytopenia [44]. The corresponding chemical structures are shown in Figure 5.

#### 2.1.6. Other Alkaloids

**Figure 6:**
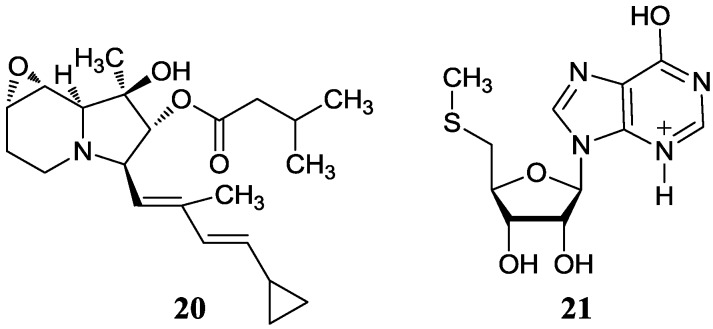
The structures of other alkaloids (**20**–**21**).

A new cyclizidine analog named JBIR-102 (**20**) was obtained from *Saccharopolyspora* sp. RL78 in mangrove soil collected in Nosoko, Ishigaki Island, Okinawa Prefecture, Japan. Compound **20** was proved to exhibit cytotoxic activities against ACC-MESO-1 and HeLa cells with IC_50_ values of 39 and 29 μM, respectively [45]. Kyeremeh and co-workers reported a novel protonated aromatic tautomer of 5’-methylthioinosine (**21**) without obvious antibacterial activity from a novel actinomycete strain *Micromonospora* sp. K310 isolated from Ghanaian mangrove river sediment in Butre, Ghana [46]. The corresponding chemical structures are shown in Figure 6.

### 2.2. Benzene Derivatives

#### 2.2.1. *p*-Aminoacetophenonic Acids

**Figure 7:**
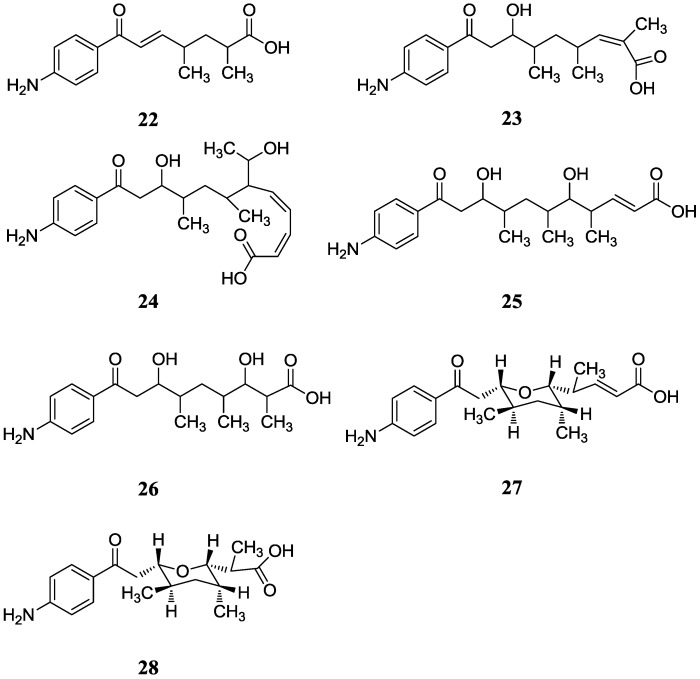
The structures of *p*-aminoacetophenonic acids (**22**–**28**).

In 2005, three new *p*-aminoacetophenonic acids (**22**–**24**) were isolated from *Streptomyces griseus* HKI0412 as an endophyte of the mangrove plant *Kandelia candel* near Xiamen City of Fujian Province, China. There was no activity research on these three compounds [47]. Then another four novel *p*-aminoacetophenonic acids (**25**–**28**) were reported in another endophyte *Streptomyces* sp. HK10552 of the mangrove plant *Aegiceras corniculatum* in the same sampling place, and these four compounds showed no cytotoxicity against the HeLa cell lines and no activity against HCV protease and SecA ATPase as well as VSVG/HIV-luc pseudotyping virus [48]. The corresponding chemical structures are shown in Figure 7.

#### 2.2.2. Benzamides

**Figure 8:**
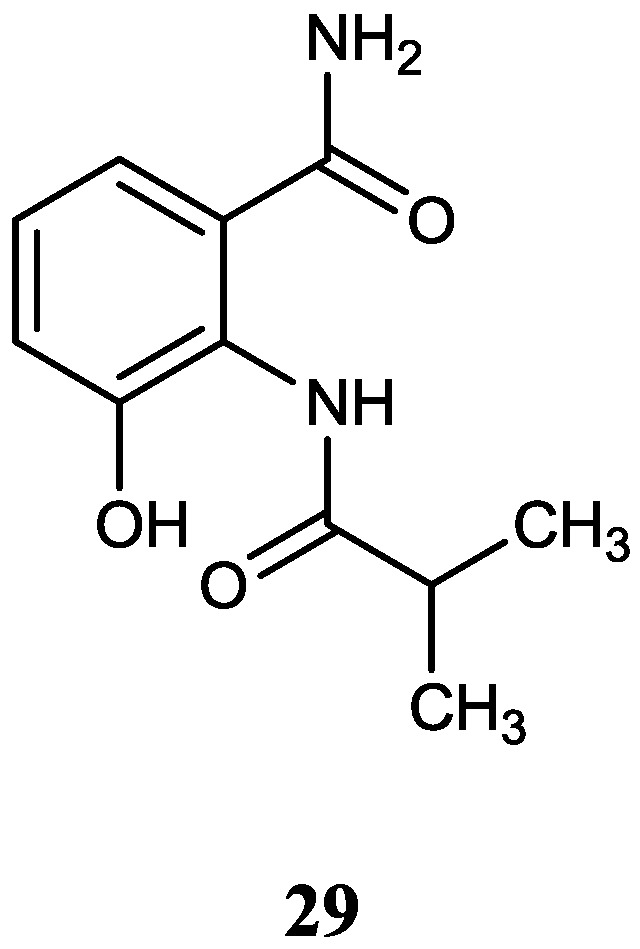
The structure of benzamide (**29**).

One new benzamide (**29**) was produced by a mangrove actinomycete *Streptomyces* sp. 061316 isolated from a mangrove soil sample in Wenchang, China [49]. Compound **29** showed no significant Caspase-3 inhibitory activity. The corresponding chemical structure is shown in Figure 8.

#### 2.2.3. Benzopyran derivatives

**Figure 9:**
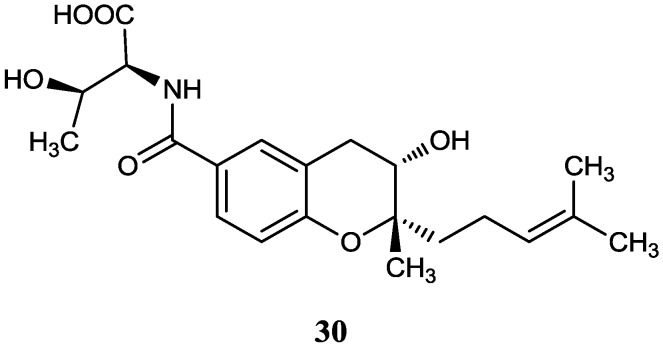
The structure of novel benzopyran derivative (**30**).

An anti-fibrotic benzopyran compound (**30**) was produced by *Streptomyces xiamenensis* 318 isolated from a mangrove sediment sample collected in the national mangrove reserve in Fujian Province of China. This compound had multiple inhibitory biological effects on lung excessive fibrotic characteristics by cell adhesion and proliferation assay at a concentration of 30 µg/mL [50]. The corresponding chemical structure is shown in Figure 9.

#### 2.2.4. Phenols

**Figure 10:**
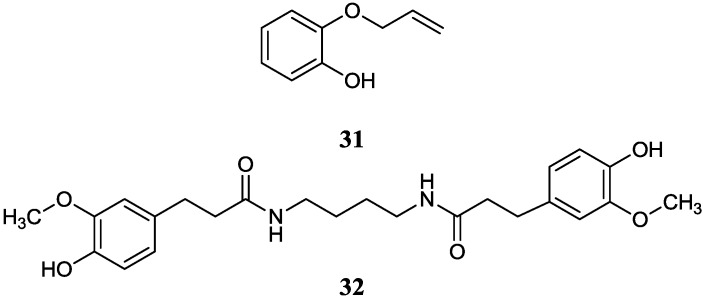
The structures of phenols (**31**–**32**).

2-Allyloxyphenol (**31**), as an important synthetic drug and chemical intermediate was obtained firstly from a new species *Streptomyces* sp. MS1/7. It was found to be an inhibitor to 21 bacteria and three fungi (*Aspergillus niger* MTCC 282, *Candida albicans* MTCC 227 and *Saccharomyces cerevisiae* MTCC 170) in the minimum range 0.2–1.75 mg/mL, and possessed strong antioxidant activity (IC_50_ = 22 μg/mL) [51]. The strain MS1/7 was isolated from sediments of the Sundarbans mangrove forest, India and the name *Streptomyces sundarbansensis* was proposed [52]. JBIR-94 (**32**) was isolated from *Streptomyces* sp. RL23 and RL66 collected from mangrove soil in Japan with antioxidative activity and it showed 1,1-diphenyl-2-picrylhydrazyl radical scavenging activity with the IC_50_ value of 11.4 μM [53,54]. The corresponding chemical structures are shown in Figure 10.

#### 2.2.5. Other Benzene Derivative

**Figure 11:**
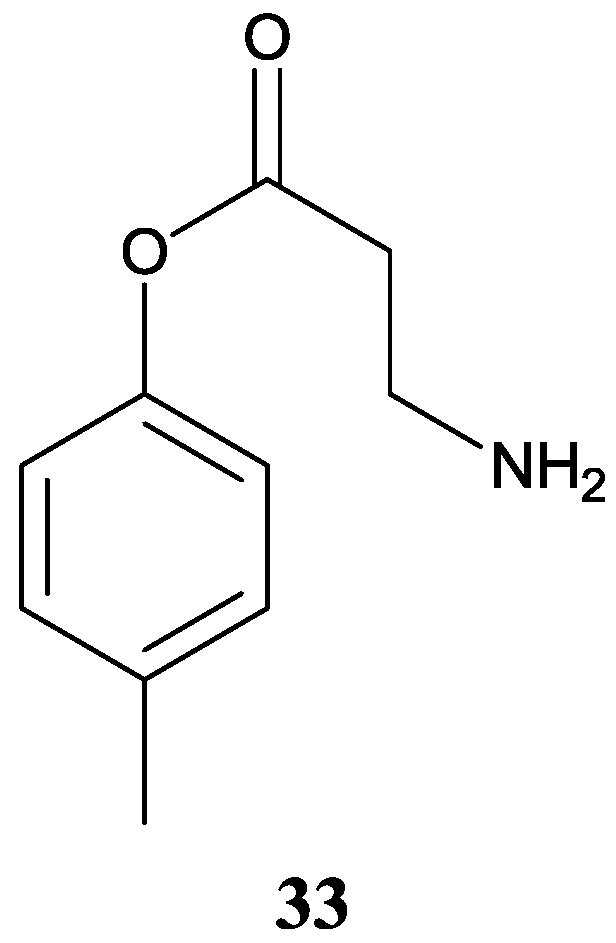
The structure of novel benzene derivative (**33**).

A novel nitrogenous compound named as *p*-tolyl-3-aminopropanoate (**33**) was produced by *Streptomyces* sp. 124092, the same strain as above and was proved to be moderately cytotoxic against SMMC-7721 with the IC_50_ value of 22 μg/mL [27]. The corresponding chemical structure is shown in Figure 11.

### 2.3. Cyclopentenone Derivatives

**Figure 12:**
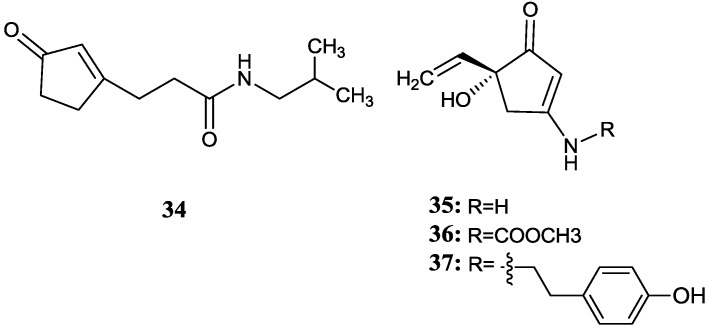
The structures of cyclopentenone derivatives (**34**–**37**).

Lin *et al.* [55] isolated four new cyclopentenone derivatives (**34**–**37**) from the endophytic *Streptomyces* sp. GT-20026114 collected from the leaves of the mangrove plant *Aegiceras comiculatum* in Xiamen, Fujian, China. However, these compounds showed no antimicrobial and antiviral activities. It is worth noting that the rare derivatives of compound **35** were isolated from a fungi previously but this is the first time the same type of novel compounds have been obtained from an actinomycete. The corresponding chemical structures are shown in Figure 12.

### 2.4. Dilactones

#### 2.4.1. Antimycins

**Figure 13:**
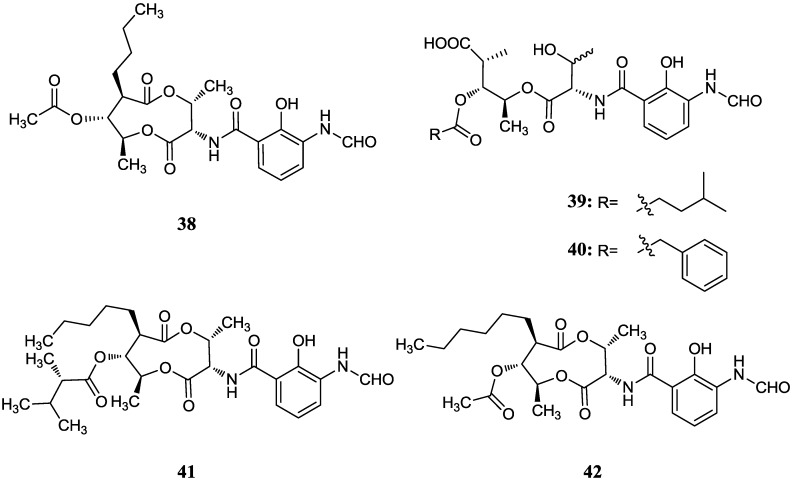
The structures of antimycins (**38**–**42**).

A series of nine-membered dilactone antimycins isolated from mangrove actinomycetes also were reported. Antimycin A18 (**38**) with a higher activity against plant pathogenic fungi than blasticidin S (a commercialized fungicide) was isolated from an endophytic *Streptomyces albidoflavus* I07A-01824 which was isolated from the leaf of *B. gymnorrhiza* collected in Shankou, Guangxi Province, China [56]. Compound **38** was regarded as the first natural antimycin which has an 8-*O*-acetyl side chain. In further study, they found that antimycin A18 also could inhibit the growth of human HepG2 and KB cancer cells (IC_50_ = 0.12 and 0.92 μg/mL), and the strain I07A-01824 was the same as a known species, *Streptomyces albidoflavus* in morphological, cultural and physiological characteristics [57]. Han *et al.* [58] then reported another two new antimycin A analogues (**39**–**40**) with the first naturally occurring open nine-membered dilactone core in antimycin A. The producer *Streptomyces lusitanus* XM52 was isolated from the rhizosphere of the mangrove plant *Avicennia mariana* from Fujian Province, China. Antimycin B1 (**39**) was inactive against the bacteria *Bacillus subtilis*, *Staphyloccocus aureus* and *Loktanella hongkongensis*, but antimycin B2 (**40**) showed antibacterial activities against *S. aureus* and *L. hongkongensis* with MIC values of 32.0 and 8.0 μg/mL respectively. Two new compounds antimycin A19 (**41**) and A20 (**42**) were isolated from *Streptomyces antibioticus* H74-18 in the bottom soil of the mangrove zone in the south China sea and these compounds showed antifungal activities against *Candida albicans* with MIC of about 5–10 μg/mL [59]. In 2011 total synthesis of the (+)-antimycin A family was reported and the nine-membered dilactone ring was obtained successfully by lactonization of a 2-pyridinethiol ester bearing a TIPS group on the 8-OH [60]. The corresponding chemical structures are shown in Figure 13.

#### 2.4.2. Other Dilactone

**Figure 14:**
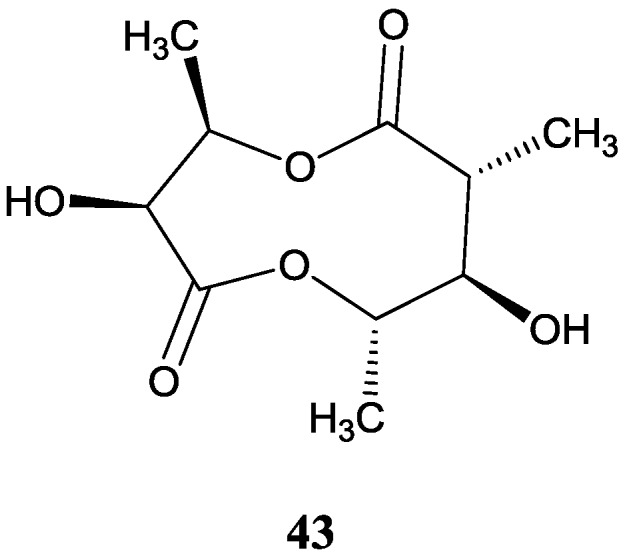
The structure of novel dilactone (**43**).

A novel dilactone (**43**) was obtained from an actinomycete strain (N2010-37) in the bottom mud of the Zhanjiang mangrove. This compound was proved to be cytotoxic against human chronic granulocytic leukemia cell line K562 *in vitro* with an IC_50_ value of 1.36 μM [61]. The corresponding chemical structure is shown in Figure 14.

### 2.5. Macrolides

#### 2.5.1. Ansa-macrolides

**Figure 15:**
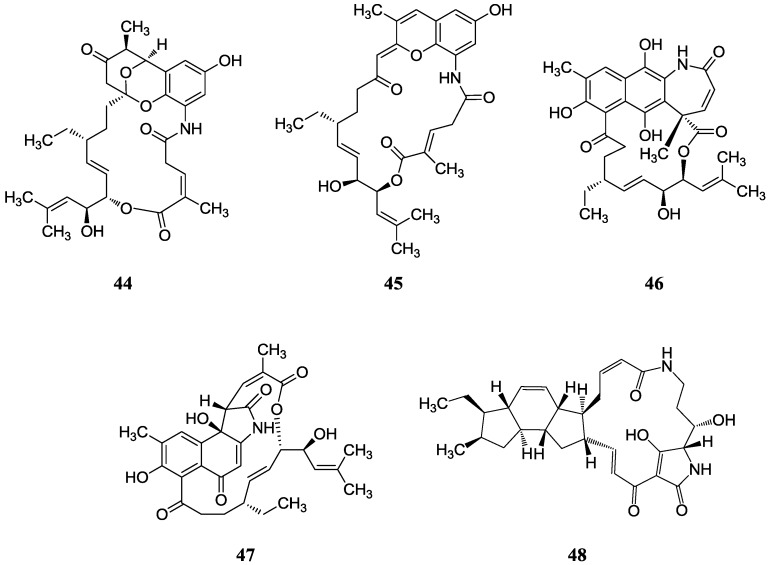
The structures of ansa-marolides (**44**–**48**).

Ding *et al.* [62] reported divergolides A-D (**44**–**47**) with complex and diverse structures isolated from an endophyte *Streptomyces* sp. HKI0576 of the mangrove tree *Bruguiera gymnorrhiza* in China. Concerning their biological activities, divergolide A (**44**) showed the strongest activity against *Mycobacterium vaccae*, whereas divergolide D (**47**) was more active against *Bacillus subtilis* and *Staphylococcus aureus* and displayed prominent activity to lung cancer (LXFA 629L), pancreatic cancer (PANC-1), renal cancer (RXF 486L), and sarcoma (Saos-2) with IC_50_ values ranging from 1.0–2.0 µM. This was the first report on the discovery of ansamycins from a tree endophyte, and the degree of “in-built diversification” of these four compounds is unprecedented for complex polyketides. Zhao *et al.* [63] reported a total synthesis of divergolide A by a ring-closing metathesis (RCM) approach in 2012. Then in 2013 Rasapalli *et al.* [64] synthesized the western segment of divergolides C (**46**) and D and established the robustness of C4-C5 as a suitable approach to further total synthesis of divergolides C and D; this chemistry was also used for divergolides A and B (**45**). A new compound butremycin (**48**) was isolated from *Micromonospora* sp. K310, the same strain as above and showed weak inhibitory activity against *S. aureus* ATCC 25923, *E. coli* ATCC 25922 and methicillin-resistant *S. aureus* (MRSA) [46]. The corresponding chemical structures are shown in Figure 15.

#### 2.5.2. Macrocyclic Dilactones

**Figure 16:**
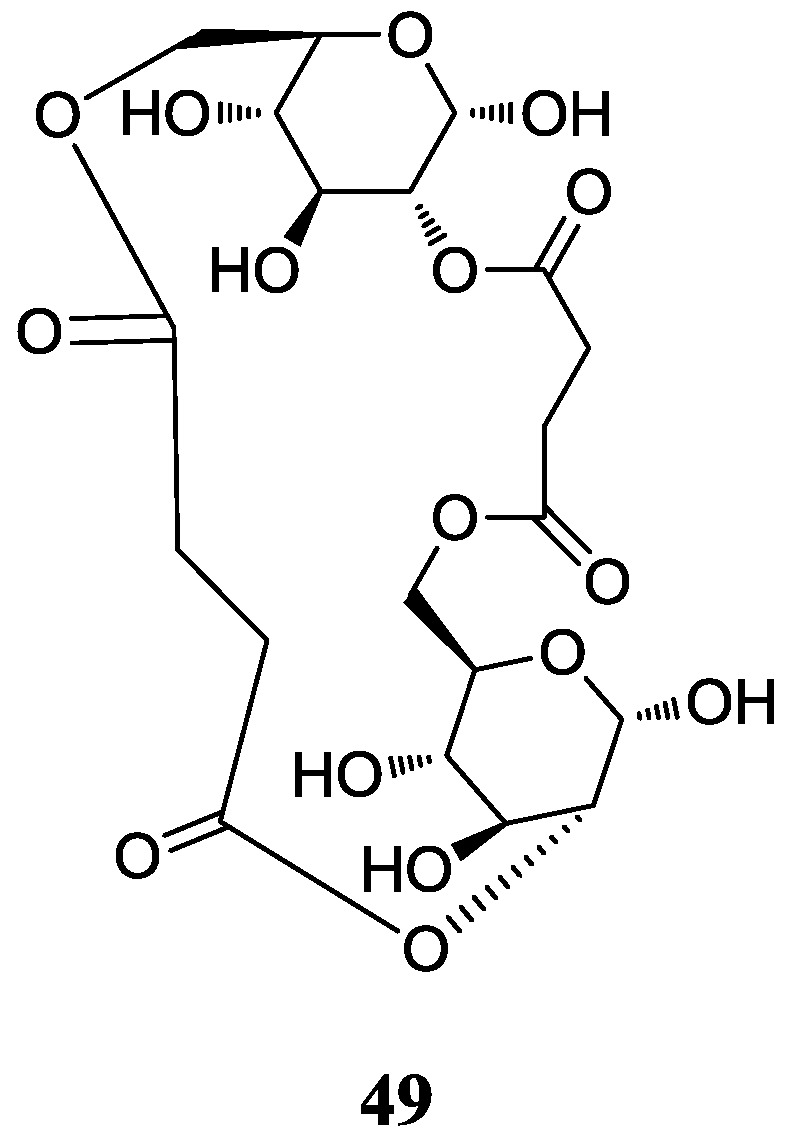
The structure of JBIR-101 (**49**).

A new macrocyclic dilactone (**49**) named as JBIR-101 with potential anti-MPM (malignant pleural mesothelioma) activity was obtained from *Promicromonospora* sp. RL26 from mangrove soil in Japan [65]. The corresponding chemical structure is shown in Figure 16.

#### 2.5.3. Macrocyclic Lactones

**Figure 17:**
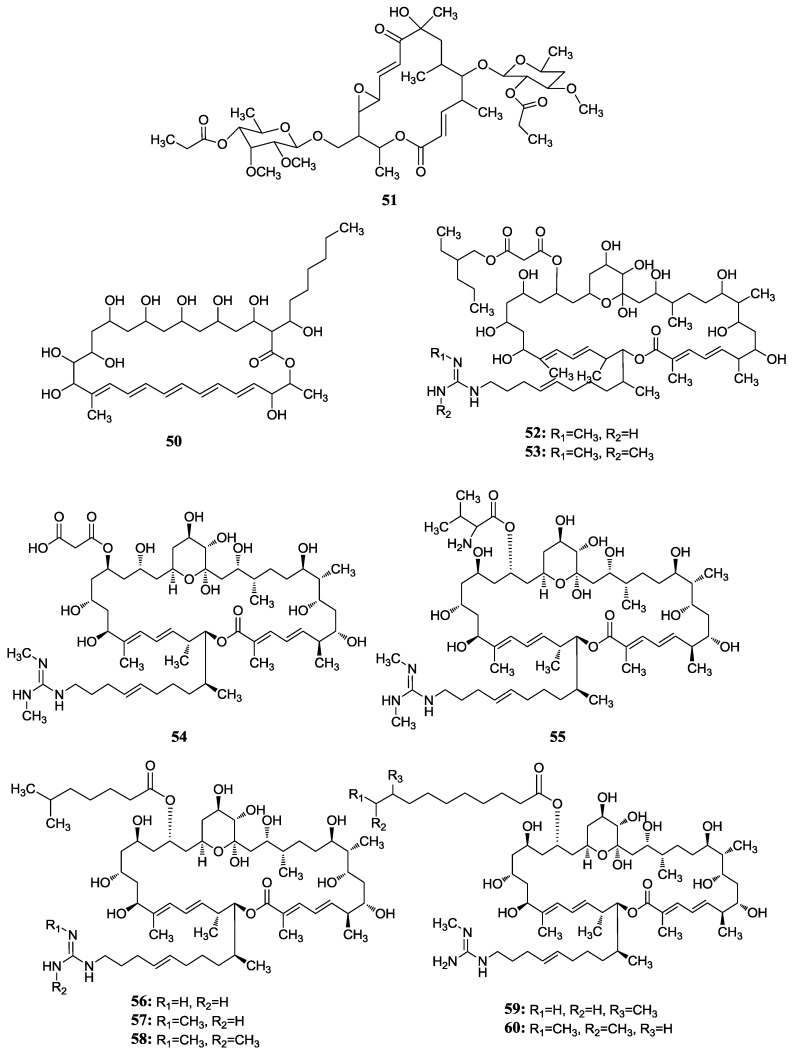
The structures of macrocyclic lactones (**54**–**60**).

A new conjugate pentaene macrolide named fungichromin B (**50**) was isolated from *Streptomyces albogriseolus* HA10002 collected from mangrove sediment in Hainan, China. Fungichromin B showed nematicidal activity against 2-stage juveniles of *M. incognita* and *M. javanica* with LD_50_ values of 7.64 and 7.83 μg/mL, and also showed a wide antifungal spectrum [66]. The first reported compound from mangrove actinomycetes was the novel chalcomycin B (**51**) isolated from the culture broth of a marine *Streptomyces* sp. B7064 which was collected from mangrove sediment near Pohoiki, Hawaii. These two macrolides showed potent activity against *Staphylococcus aureus* (MIC = 0.39 μg/mL), *Bacillus subtilis* (MIC = 6.25 μg/mL) and antimicrobial activity to *Chlorella vulgaris*, *Chlorella sorokiniana* and *Scenedesmus subspicatus* at the MIC of 50 μg/mL [67]. Two new macrocyclic lactones were identified as azalomycin F4a 2-ethylpentyl ester (**52**) and azalomycin F5a 2-ethylpentyl ester (**53**) by Yuan *et al.* [68]. They were isolated from the broth of mangrove *Streptomyces* sp. 211726 which was isolated from mangrove rhizosphere soil of *Heritiera globosa* collected in Wenchang, China. Compounds **52** and **53** showed activity against *Candida albicans* ATCC 10231 at the MIC of 2.34 and 12.5 μg/mL and moderate cytotoxicity against HCT-116 cell line with IC_50_ values of 5.64 and 2.58 μg/mL. Recently, another seven new azalomycin F analogs (**54**–**60**) were identified from this strain with the same fermentation condition and showed broad-spectrum antimicrobial activity and moderate cytotoxicity against HCT-116 *in vitro* with IC_50_ values of 1.81–5.00 μg/mL [69]. Moreover, these seven compounds also exhibited broad-spectrum antimicrobial activity against *Candida albicans* ATCC 10231, *Staphylococcus aureus* S014, *Bacillus subtilis* S028 and *Escherichia coli* S002. The corresponding chemical structures are shown in Figure 17.

#### 2.6. 2-Pyranones Derivatives

**Figure 18:**
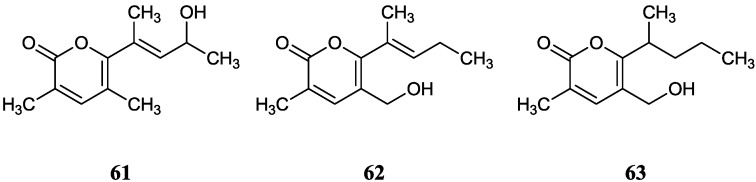
The structures of 2-pyranone derivatives (**61**–**63**).

In 2010, three new 2-pyranone derivatives, namely norcardiatones A (**61**), B (**62**) and C (**63**) were isolated from the agar cultures of the strain *Nocardiopsis* sp. A00203 which was isolated from the leaves of *Aegiceras corniculatum* in Jimei, Fujian, China [70]. However, they had no inhibitory effect on *Escherichia coli*, *Bacillus subtilis*, *Staphylococcus aureus* and yeasts at a concentration of 50 µg/disc and only norcardiatone A showed weak cytotoxicity against HeLa cells (10 µg/mL, 2.73% and 20 µg/mL, 7.39%). The corresponding chemical structures are shown in Figure 18.

### 2.7. Sesquiterpenes

#### 2.7.1. Eudesmene-Type Sesquiterpenes

**Figure 19:**
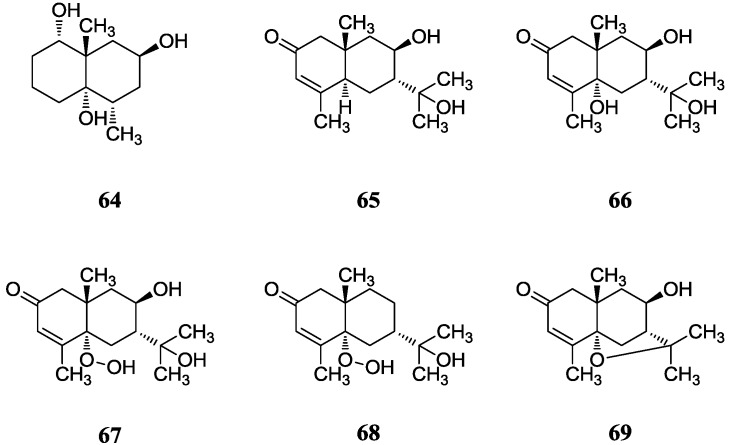
The structure of eudesmene-type sesquiterpene (**64**–**69**).

A new degraded sesquiterpene (**64**) with a eudesmane-type skeleton was obtained from *Streptomyces* sp. 0616208 which was isolated from mangrove sediment in the South China Sea [71]. Its structure was elucidated as (1α, 4aα, 5α, 7β, 8aβ)-5, 8a-dimethyldecahydrona-phthalene-1, 4a, 7-triol and compound **64** showed moderate cytotoxicity against human hepatoma SMMC-7721 cell line. Kandenols A–E (**65**–**69**) were reported by Ding *et al.* in 2012 [72]. These novel compounds were isolated from *Streptomyces* sp. HKI0595 which was the same producer of xiamycins and exhibited no cytotoxicity against 12 human cell lines, and weak antimicrobial activities against *Bacillus subtilis* ATCC 6633 and *Mycobacterium vaccae* IMET 10670. Compound **69** was believed to be the first bacterial agarofuran, while compounds **67** and **68** with hydroperoxide groups were also unusual. Notably, the family of eudesmanes are secondary metabolites mainly from plants. The corresponding chemical structure is shown in Figure 19.

#### 2.7.2. Germacrane-Type Sesquiterpenes

**Figure 20:**
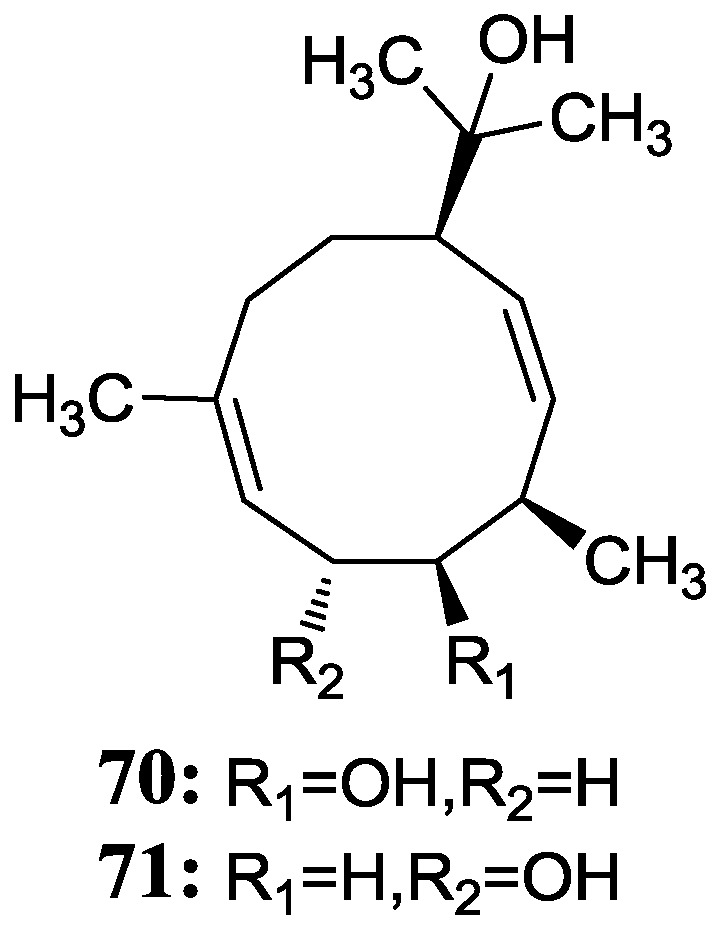
The structures of germacrane-type sesquiterpenes (**70**–**71**).

Guan *et al.* [73] isolated two novel germacrane-type sesquiterpene alcohols (**70**–**71**) without bioactivity research reports from a subspecies of *Streptomyces griseus* collected from an endophyte of the mangrove plant *Kandelia candel* in Xiamen city of Fujian Province, China. The corresponding chemical structures are shown in Figure 20.

### 2.8. Miscellaneous

#### 2.8.1. 2-Acylglycerol Derivatives

**Figure 21:**
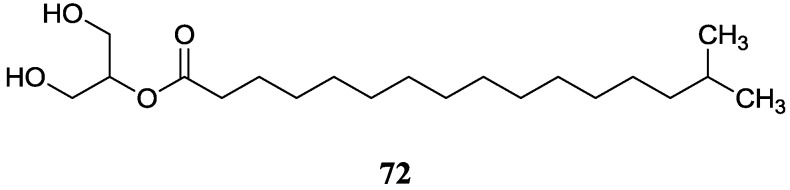
The structure of 2-acylglycerol derivative (**72**).

A new compound named 15-methyl hexadecanoic acid 2-acylglycerol (**72**) was discovered in *Streptomyces* sp. 211726, the same strain as above [74]. Compound **72** showed no obvious cytotoxicity against HCT-116 cell line (IC_50_ > 20 µg/mL). The corresponding chemical structure is shown in Figure 21.

#### 2.8.2. Flavones

**Figure 22:**
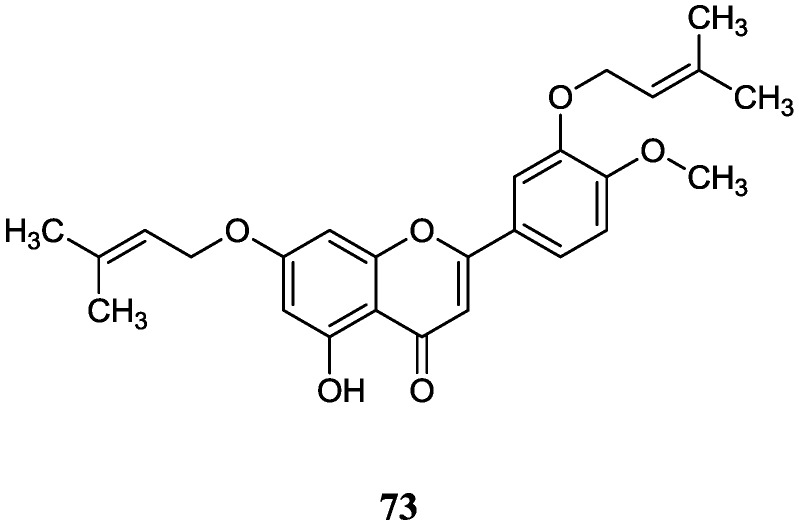
The structure of novel flavone (**73**).

A new di-*O*-prenylated flavone (**73**) was obtained from *Streptomyces* sp. MA-12 isolated from the root of the semi-mangrove plant *Myoporum bontioides* A collected in Leizhou Peninsula, Guangdong Province, China. It showed moderate inhibitory activity against three plant pathogenic fungi including *Colletotrichum musae*, *Gibberella zeae* and *Penicillium citrinum* at a concentration of 0.25 mM [75]. The corresponding chemical structure is shown in Figure 22.

Inevitably, known compounds have also been isolated from mangrove actinomycetes. Table 1 represents these various known compounds and their bioactivity. Meanwhile, chemical structures and respective sources are also listed.

**Table 1:** Known secondary metabolites isolated from mangrove actinomycetes.

Compound	Isolate	Source	Bioactivity	Reference
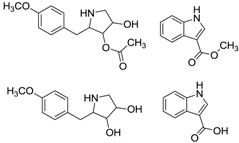	*Streptomyces* sp. 060524	Mangrove seaweed (Haikou, China)	Antitumor	[76]
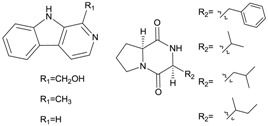	*Micromonospora* sp. M2DG17	Composite mangrove sediment (Haikou, China)	Antitumor	[77,78]
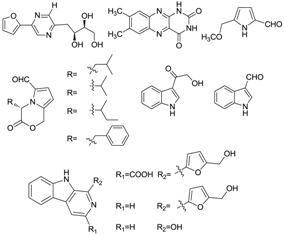	*Jishengella endophytica* 161111	Mangrove rhizosphere soil and root (Hainan, China)	Anti-H1N1 virus	[36,37]
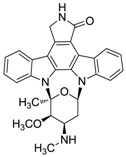	*Streptomyces* sp. 172614	Mangrove soil (Fujian, China)	Antitumor	[33]
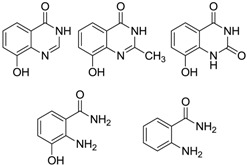	*Streptomyces* sp. 061316	Mangrove soil (Wenchang, China)	Caspase-3 inhibitory activity	[49]
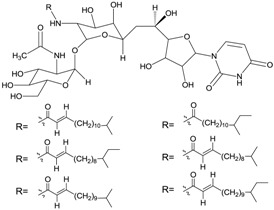	Marine actinomycete strain H83-3	Mangrove bottom mud (South China Sea)	Antifungal	[79]
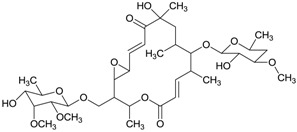	*Streptomycete* sp. B7064	Mangrove sediment (Pohoiki, Hawaii)	Antimicrobial	[67]
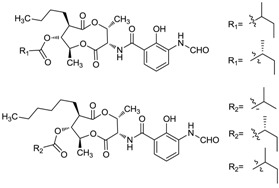	*Streptomyces antibioticus* H74-18	Mangrove bottom soil (South China Sea)	Antifungal	[59]
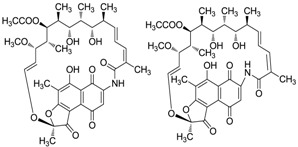	*Micromonospora rifamycinica* AM105	Mangrove sediment (South China Sea)	Antibacterial	[80]
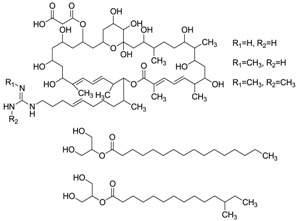	*Streptomyces* sp. 211726	Mangrove rhizosphere soil (Wenchang, China)	Antimicrobial Antitumor	[68]
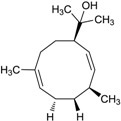	*Streptomyces griseus* subsp.	Mangrove plant stems (Xiamen, China)	Unknown	[73]

## 3. Concluding Remarks and Future Perspectives

Mangrove actinomycetes as productive microbial communities are becoming a hot spot for natural products studies. Until the time of writing, 122 various secondary metabolites including 73 new and 49 known compounds isolated from mangrove actinomycetes had been collected including alkaloids, benzene and cyclopentenone derivatives, dilactones, macrolides, 2-pyranones, sesquiterpenes and some attractive structures such as salinosporamides, xiamycins and novel indolocarbazoles which have been highlighted. Although some known compounds have been isolated repeatedly, they have been proven to show potential novel bioactivity together with the adoption of more and more new bioactivity screenings and tests. Macrolides, alkaloids and sesquiterpenes form the majority of these significant compounds with alkaloids being the most. It has been observed that the genus *Streptomyces* among mangrove actinomycetes as natural product producers is the richest source followed by the genus *Micromonospora*. Indol alkaloids including indolosesquiterpenes and indolocarbazoles, macrolides and benzene derivatives are the main natural products of the genus *Streptomyces*. Alkaloids and sesquiterpenes are the main secondary metabolites of the genus *Micromonospora*. Alkaloids are the main natural products of two novel genus *Jishengella* and *Salinispora* and therein salinosporamides with specific structures have received significant attention. Obviously, the study on mangrove actinomycetes and their secondary metabolites is just beginning and mangrove actinomycetes require urgent exploitation as a valuable source of natural products. The use of novel strategies such as screening model building, sequence-based screening, genome mining or selective isolation from new species may improve the efficiency of discovering oruginal natural products from mangrove actinomycetes.

Although the isolation of natural products from mangrove actinomycetes has given some inspiring and gratifying results, challenges still exist for the following reasons: (1) frequent rediscovery of known natural products; (2) technical challenges associated with their purification and structural identification; (3) the continued decline of the mangroves and complete disappearance of the mangroves over the next 100 years according to the present rate of loss [7]; (4) the not applicable traditional screening strategies at breaking through the existing bottleneck and the not yet mature novel screening strategies which retain some limitations; (5) vast uncultivable environmental microbes as an unexploited source of natural products; (6) the financial cost of new natural product discovery. There still remains a lot of work to unearth the potential of mangrove actinomycetes as natural product producers. If the current limitations of technologies or strategies could be gradually conquered, there will be a bright future for natural products from mangrove actinomycetes as novel marine drugs for the benefit of human health.
